# Cardiovascular disease preventive effects of aspirin combined with different statins in the United States general population

**DOI:** 10.1038/s41598-023-31739-w

**Published:** 2023-03-20

**Authors:** Tao Liu, Ronghua Zuo, Jia Wang, Zixuan Huangtao, Bing Wang, Lifang Sun, Shasha Wang, Baoyin Li, Zhijian Zhu, Yesheng Pan

**Affiliations:** 1 Department of Cardiology, Jinshan Branch of Shanghai Sixth People’s Hospital, Shanghai, 201500 China; 2 Department of Anesthesiology, The First Affiliated Hospital of Nanjing Medical University, Nanjing, 210029 Jiangsu China; 3 Department of Nephrology, The Affiliated Hospital of Xuzhou Medical University, Xuzhou, 221000 Jiangsu China; 4 School of Clinical Medicine, Hainan Medical University, Haikou, 57119 Hainan China

**Keywords:** Cardiology, Health care, Medical research

## Abstract

The purpose of this study was to explore the use of aspirin in conjunction with various statins for cardiovascular disease (CVD) prevention in the general population of the United States (U.S.). A total of 3778 people from the National Health and Nutrition Examination Surveys from 2011 to 2018 were included in our analysis. After adjusting for sociodemographic and common cardiovascular risk factors, we used multivariable logistic regression analysis to determine aspirin should be combined with which type of statin for better CVD preventive effects. Subgroup analyses were carried out subsequently. In comparison to the aspirin use alone, the odds ratios with 95% confidence intervals for CVD were 0.43 (0.33, 0.57), 0.69 (0.42, 1.13), 0.44 (0.31, 0.62), 0.34 (0.23, 0.50) and 0.64 (0.49, 0.84) for the combination use of aspirin and atorvastatin, lovastatin, pravastatin, rosuvastatin as well as simvastatin, respectively, in the fully-adjusted model. Aspirin combined with rosuvastatin was more effective in the prevention of individual CVD, including congestive heart failure, coronary heart disease, angina pectoris and heart attack, than aspirin combined with other statins. In conclusion, statins combined with aspirin have a clear advantage over aspirin alone in preventing CVD. In addition, when various sex, age, and fitness levels were considered, as well as with and without diabetes mellitus, the combination usage of aspirin and rosuvastatin had the greatest CVD preventive effects than aspirin coupled with other statins.

## Introduction

Cardiovascular disease (CVD), including atherosclerosis, heart failure, cerebrovascular disease, peripheral vascular disease and other cardiac abnormalities, is the leading cause of death worldwide, and both its prevalence and fatality rate are increasing^1^. Every year, approximately 655,000 Americans die as a result of cardiovascular disease, resulting in a massive economic burden^2^.

Aspirin, a nonsteroidal anti-inflammatory drug, has been shown to inhibit the formation of various inflammatory mediators and adhesion molecules, resulting in anti-atherosclerosis^3,4^. Aspirin reduces the risk of major vascular events by 15% to 20% when used for primary CVD prevention^5^. As a result, the role of aspirin in the prevention and treatment of CVD is widely acknowledged in the medical community^6^. HMG-CoA reductase enzyme is the starting point of cholesterol synthesis, and statins compete with the enzyme and thus impede the beginning of cholesterol production^7^. Statins, a class of lipid-lowering drugs, have a substantial impact on decreasing lipids and delaying plaque development, thus lowering morbidity and death in individuals with atherosclerotic CVD^8^. Statin therapy has been shown to reduce the risk of cardiovascular events by 30% to 40% when used for primary prevention of CVD^5^. Therefore, in the prevention of primary CVD, the combination of statins with aspirin is more beneficial than aspirin alone.

However, it is unclear which combination of aspirin and statins provides the greatest protection against cardiovascular events. Therefore, we used the data from the National Health and Nutrition Examination Survey (NHANES) database to investigate which type of statin, when combined with aspirin, has the best effect for CVD prevention.

## Materials and methods

### Study population

The NHANES database is a complex survey that combines interviews and physical examinations to obtain a nationally representative sample of the civilian, noninstitutionalized United States (U.S.) population (https://www.cdc.gov/nchs/nhanes/)^9^. In this study, we analyzed NHANES data from four 2-year cycles (2011–2018). Participants with missing CVD and information on the aspirin and statin medication questionnaires (N = 16,090 and 16,449, respectively) were excluded. Finally, a total of 3378 participants took part in this study. Because the current study relied on existing data from the NHANES database and did not involve the collection of new data, no ethical approvals were required. The National Center for Health Statistics Institutional Review Board approved all NHANES procedures, and all participants provided written informed consent^10^. The NHANES website (https://www.cdc.gov/nchs/nhanes/) contains complete information about the survey design, methodology, and data.

### Aspirin and statin use

The use of aspirin and various statins was determined using data from the standardized NHANES 2011–2018 questionnaire. To determine the use of aspirin, participants were asked the following questions: “Do your doctors or other health care providers advise you to take a low-dose aspirin every day to prevent heart attacks, strokes, or cancer? Have you ever been told to do this?”, “Are you now doing this?”, and “Are you taking a low-dose aspirin every day on your own to prevent heart attacks, strokes, or cancer?” Using these questionnaires, a binary variable (yes or no) was created to indicate participants’ current aspirin use. In terms of statin use, participants were asked if they had used any medications that required a prescription in the previous month. Those who said yes were then asked to show the investigator the medication containers for all of the products they had used. Statins with the generic name’s atorvastatin, lovastatin, pravastatin, rosuvastatin, simvastatin, and combination products were studied. The NHANES website (https://wwwn.cdc.gov/nchs/nhanes/Default.aspx) contains detailed information and procedures.

### Covariates

The following covariates were downloaded from the NHANES database: age, sex, race/ethnicity, family poverty income ratio (PIR), education level, marital status, the history of hypertension, and diabetes mellitus (DM), smoker, alcohol user, body mass index (BMI), physical activity (PA), mean energy intake, hemoglobin (HB), high-density lipoprotein-cholesterol (HDL-C), total cholesterol (TC), triglyceride (TC), blood urea nitrogen (BUN), uric acid (UA), serum creatinine (Scr). During the home interview, the following data were self-reported by the participants: age, sex, race/ethnicity, education level, marital status, smoking status, drinking status, and mean energy intake. Sex was dichotomized into two groups (male and female). Education was categorized into five groups (< 9th grade, 9–11th grade, high school, college, and graduate). Self-identified race/ethnicity was grouped as Mexican American, Other Hispanic, Non-Hispanic White, Non-Hispanic Black, and Other race. Marital status was categorized into three groups (married, separated, and never married). Family poverty-to-income was defined as the total family income divided by the poverty threshold^11^. In addition, data on Hb, HDL-C, TC, TG, BUN, UA, and Scr were obtained from laboratory tests. Frequency of aspirin use includes ‘one every day of aspirin’, and ‘one every other day of aspirin’. Details of all variables are available online at https://www.cdc.gov/nchs/nhanes/.

### CVD ascertainment

The primary outcome for the study was CVD which defined as a composite of five self-reported outcomes (congestive heart failure (CHF), coronary heart disease (CHD), angina pectoris, heart attack and stroke)^12^. The participant was recorded as having CVD if she/he answered “yes” to the following question: “Has a doctor or other health professional ever told you that you had congestive heart failure/coronary heart disease/angina pectoris/stroke?”. A standardized medical condition questionnaire administered during the personal interview provides more detailed information (www.cdc.gov/nchs/nhanes/). Primary prevention was defined in this study as the prevention of the first occurrence of a cardiovascular event, such as self-reported CHD, CHF, angina/angina pectoris, heart attack, or stroke.

### Statistical analysis

Continuous variables were presented as means standard deviation and categorical variables as frequency or percentage. Furthermore, we built three models: model 1, which adjusted for age and sex; model 2, which adjusted for age, sex, race, educational level, marital status, family PIR, smoker, alcohol user, hypertension, DM, and BMI; and model 3, which adjusted for all of the potential confounding factors listed in Table 1. The multivariable logistic regression models were used to investigate the use of aspirin in combination with various statins for primary CVD prevention. The stratified and interaction models were used to perform subgroup analysis. All the analyses were performed with R version 3.6.4 (R Foundation for Statistical Computing, Vienna, Austria), SPSS version 22.0 (SPSS Inc., Chicago, IL, USA) and EmpowerStats software (http://www.empowerstats.com). *P-*value < 0.05 was regarded as statistically significant.

**Table 1:** Demographic characteristics of the study participants.

Variables	Overall (n = 3778)	CVD (n = 2520)	Non-CVD (n = 1258)	*P*-value
Age, years	66.45 ± 10.22	67.58 ± 9.99	63.75 ± 10.04	< 0.001
Gender, %				< 0.001
Male	2026 (53.6%)	1267 (33.5%)	759 (20.1%)	
Female	1752 (46.4%)	1253 (33.2%)	499 (13.2%)	
Race, %				< 0.001
Mexican American	381 (10.1%)	284 (7.5%)	97 (2.6%)	
Other Hispanic	343 (9.1%)	247 (6.5%)	96 (2.5%)	
Non-Hispanic White	1746 (46.2%)	1112 (29.4%)	634 (16.8%)	
Non-Hispanic Black	901 (23.8%)	606 (16.0%)	295 (7.8%)	
Other race	407 (10.8%)	271 (7.2%)	136 (3.6%)	
Family poverty-income ratio	2.54 ± 1.61	2.64 ± 1.60	3.17 ± 1.65	< 0.001
Education level, %				< 0.001
< 9th grade	645 (17.1%)	386 (10.2%)	259 (6.9%)	
9–11th grade	746 (19.7%)	467 (12.3%)	279 (7.4%)	
High school	1425 (37.7%)	958 (25.3%)	467 (12.4%)	
College	525 (13.9%)	373 (9.9%)	152 (4.0%)	
Graduate	437 (11.6%)	336 (8.9%)	101 (2.7%)	
Marital status, %				< 0.001
Having a partner	2270 (60.1%)	1555 (41.2%)	715(18.9%)	
No partner	1383 (36.6%)	866 (22.9%)	517 (13.7%)	
Unmarried	125 (3.3%)	99 (2.6%)	26 (0.7%)	
Hypertension, %				< 0.001
No	1050 (27.8%)	810 (21.4%)	240 (6.4%)	
Yes	2728 (72.2%)	1710 (45.3%)	1018 (26.9%)	
DM, %				< 0.001
No	2423 (64.1%)	1682 (44.5%)	741 (19.6%)	
Yes	1355 (35.9%)	838 (22.2%)	517 (13.7%)	
Smoker, %				< 0.001
No	1834 (48.5%)	1327 (35.1%)	507 (13.4%)	
Yes	1944 (51.5%)	1193 (31.6%)	751 (19.9%)	
Alcohol user, %				0.319
No	1018 (26.9%)	683 (18.1%)	335 (8.9%)	
Yes	2760 (73.1%)	1837 (48.6%)	923 (24.4%)	
PA, %				0.913
Vigorous	564 (14.9%)	381 (10.1%)	183 (14.5%)	
Moderate	788 (20.9%)	520 (13.8%)	268 (7.1%)	
Never	2426 (64.2%)	1619 (42.9%)	807 (21.4%)	
BMI, kg/m^2^	30.31 ± 6.74	30.50 ± 6.62	30.41 ± 6.60	0.684
Mean energy intake (kcal/day)	1880.75 ± 721.71	1919.57 ± 730.93	1997.12 ± 706.79	0.006
Hb, g/dL	13.80 ± 1.51	13.92 ± 1.60	14.13 ± 1.33	< 0.001
BUN, mg/dL	17.07 ± 7.57	18.09 ± 7.86	16.19 ± 5.89	< 0.001
UA, mg/dL	5.77 ± 1.48	5.92 ± 1.55	5.58 ± 1.36	< 0.001
Scr, mg/dL	1.03 ± 0.65	1.05 ± 0.50	0.93 ± 0.49	< 0.001
HDL-cholesterol, mg/dL	52.20 ± 15.93	49.83 ± 16.46	54.56 ± 17.60	< 0.001
TC, mg/dL	182.63 ± 45.11	171.14 ± 43.87	192.99 ± 43.35	< 0.001
TG, mg/dL	163.76 ± 118.50	167.58 ± 112.94	167.63 ± 124.16	0.992
CVD medications, %				< 0.001
Aspirin alone	1587 (42.0%)	1245 (33.0%)	342 (9.0%)	
Atorvastatin and aspirin combination	778 (20.6%)	425 (11.3%)	353 (9.3%)	
Lovastatin and aspirin combination	135 (3.6%)	88 (2.4%)	47 (1.2%)	
Pravastatin and aspirin combination	290 (7.7%)	169 (4.5%)	121 (3.2%)	
Rosuvastatin and aspirin combination	233 (6.2%)	120 (3.2%)	113 (3.0%)	
Simvastatin and aspirin combination	755 (20.0%)	473 (12.5%)	282 (7.5%)	
Frequency of aspirin use, %				< 0.001
One every day	3543 (93.8%)	2323 (61.5%)	1220 (32.3%)	
One every other day	235 (6.2%)	197 (5.2%)	38(1.0%)	
Aspirin dose, mg	111.51 ± 82.38	128.52 ± 96.73	108.81 ± 80.87	< 0.001

Data are presented as mean ± SD or n (%).

*CVD* cardiovascular disease, *DM* diabetes mellitus, *PA* physical activity, *BMI* body mass index, *Hb* hemoglobin, *Scr* serum creatinine, *BUN* blood urea nitrogen, *UA* uric acid, *TC* total cholesterol, *TG* triglycerides, *HDL-cholesterol* high density lipoprotein-cholesterol.

### Ethical approval and consent to participate

All NHANES participants provided written informed consent and the National Center for Health Statistics obtained institutional review board approval prior to data collection. Because NHANES data are de-identified and publicly available, the analysis presented here was exempt from IRB review.

## Results

### Baseline characteristics

Table 1 shows the baseline characteristics of the study participants. In total, 3778 people (aged 66.45 ± 10.22 years) were included in our study. According to weighted analysis, the number of people included in our study represents the overall U.S. population of 2,468,896. CVD was present in 27.7% of this population. There were significant differences in baseline characteristics between the CVD group and non-CVD group, with the exception of the alcohol user, PA, BMI, and TG.

### Association between aspirin and different statins use and total CVD

Table 2 shows the results of multivariable logistic regression analyses for the association between aspirin and different statin use and total CVD. After controlling for underlying cofounders, the odds ratios (ORs) with 95% confidence intervals (CIs) for CVD were 0.43 (0.33, 0.57), 0.69 (0.42, 1.13), 0.44 (0.31, 0.62), 0.34 (0.23, 0.50) and 0.64 (0.49, 0.84) for aspirin and different statin combinations (atorvastatin, lovastatin, pravastatin, rosuvastatin and simvastatin).

**Table 2:** Associations of aspirin alone compared to aspirin plus statin with the risk of total CVD.

CVD medications	Model 1	Model 2	Model 3
	OR (95% CI)	OR (95% CI)	OR (95% CI)
Aspirin alone	Ref.	Ref.	Ref.
Atorvastatin and aspirin combination	0.36 (0.30, 0.44)***	0.36 (0.28, 0.45)***	0.43 (0.33, 0.57)***
Lovastatin and aspirin combination	0.55 (0.38, 0.80)**	0.65 (0.42, 1.01)	0.69 (0.42, 1.13)
Pravastatin and aspirin combination	0.40 (0.31, 0.52)***	0.43 (0.31, 0.58)***	0.44 (0.31, 0.62)***
Rosuvastatin and aspirin combination	0.31 (0.23, 0.41)***	0.34 (0.25, 0.48)***	0.34 (0.23, 0.50)***
Simvastatin and aspirin combination	0.53 (0.43, 0.64)***	0.54 (0.43, 0.68)***	0.64 (0.49, 0.84)**

Model 1: age and gender. Model 2: Model 1 variables plus education level, race/ethnicity, family poverty-income ratio, hypertension, diabetes mellitus, smoker, alcohol user, and body mass index. Model 3 was adjusted for Model 2 variables plus physical activity, total energy intake, high-density lipoprotein-cholesterol, total cholesterol, triglyceride, uric acid, creatinine, blood urea nitrogen, and hemoglobin.

*CVD* cardiovascular disease, *OR* odd ratio, *CI* confidence interval.

**P < 0.01, ***P < 0.001.

### Association between aspirin and different statins use and individual CVDs

Separate analyses were conducted to examine the relationship between aspirin and various statins use and individual CVDs such as CHF, CHD, angina pectoris, heart attack, and stroke. The findings revealed that there was a strong link between the use of aspirin and different statins and the prevalence of individual CVDs such as CHD, CHF, angina pectoris, and heart attack. The ORs (95% CIs) of individual CVDs were 0.47 (0.27, 0.84), 0.24 (0.15, 0.39), 0.24 (0.13, 0.42), 0.30 (0.19, 0.49), and 0.98 (0.54, 1.81) in the fully adjusted model for rosuvastatin and aspirin combination, respectively, compared to aspirin use alone (Table 3).

**Table 3:** Associations of aspirin alone compared to aspirin plus statin with the risk of individual CVD.

CVD medications	CHF	CHD	Angina	Heart attack	Stroke
	OR (95% CI)	OR (95% CI)	OR (95% CI)	OR (95% CI)	OR (95% CI)
Aspirin alone	1.00	1.00	1.00	1.00	1.00
Atorvastatin and aspirin combination	0.51 (0.34, 0.77)**	0.28 (0.19, 0.39)***	0.37 (0.23, 0.59)***	0.37 (0.26, 0.54)***	0.90 (0.60, 1.35)
Lovastatin and aspirin combination	0.91 (0.39, 2.13)	0.50 (0.26, 0.96)*	0.64 (0.26, 1.60)	0.74 (0.368, 1.482)	0.63 (0.33, 1.22)
Pravastatin and aspirin combination	0.60 (0.36, 1.02)	0.38 (0.24, 0.60)***	0.34 (0.19, 0.60)***	0.47 (0.297, 0.749)***	0.58 (0.36, 0.92)*
Rosuvastatin and aspirin combination	0.47 (0.27, 0.84)*	0.24 (0.15, 0.39)***	0.24 (0.13, 0.42)***	0.30 (0.189, 0.488)***	0.98 (0.54, 1.81)
Simvastatin and aspirin combination	0.65 (0.42, 0.99)*	0.40 (0.28, 0.57)***	0.42 (0.26, 0.67)***	0.48 (0.334, 0.685)***	1.07 (0.71, 1.62)

Analysis was adjusted for age, gender, education level, race/ethnicity, marital status, family poverty-income ratio, hypertension, diabetes mellitus, smoker, alcohol user, body mass index, physical activity, total energy intake, high-density lipoprotein-cholesterol, total cholesterol, triglyceride, uric acid, creatinine, blood urea nitrogen, and hemoglobin.

*CVD* cardiovascular disease, *CHF* congestive heart failure, *CHD* coronary heart disease, *OR* odd ratio, *CI* confidence interval.

*P < 0.05, **P < 0.01, ***P < 0.001  .

### Subgroup analyses

Subgroup analyses were carried out based on age, sex, hypertension, DM, and BMI (Table 4). With respect to the subgroup analyses (age, sex, DM, and BMI), rosuvastatin combined with aspirin was shown to be more effective for the prevention of CVD. However, in populations without hypertension, the combination of atorvastatin plus aspirin was more effective in preventing CVD. Furthermore, there were significant differences in the use of different statins and aspirins in preventing CVD in terms of age, hypertension, and DM.

**Table 4:** Subgroups analysis for the associations of aspirin alone compared to aspirin plus statin with the prevalence of total CVD.

	Aspirin alone	Atorvastatin and aspirin combination	Lovastatin and aspirin combination	Pravastatin and aspirin combination	Rosuvastatin and aspirin combination	Simvastatin and aspirin combination	*P* for interaction
	OR (95% CI)	OR (95% CI)	OR (95% CI)	OR (95% CI)	OR (95% CI)	OR (95% CI)	
Sex							0.126
Male	1.00	0.32 (0.22, 0.46)***	0.55 (0.28, 1.07)	0.36 (0.22, 0.59)***	0.21 (0.12, 0.37)***	0.44 (0.31, 0.64)	
Female	1.00	0.59 (0.39, 0.89)*	0.81 (0.38, 1.73)	0.51 (0.31, 0.83)**	0.50 (0.28, 0.89)*	0.98 (0.64, 1.49)	
Age							< 0.001
< 60	1.00	0.41 (0.23, 0.74)**	0.94 (0.24, 3.74)	0.28 (0.14, 0.57)***	0.23 (0.10, 0.52)***	0.68 (0.35, 1.33)	
≥ 60	1.00	0.44 (0.32, 0.60)***	0.65 (0.38, 1.11)	0.51 (0.34, 0.80)**	0.38 (0.24, 0.60)***	0.63 (0.47, 0.85)**	
Hypertension							< 0.001
No	1.00	0.27 (0.15, 0.47)***	0.41 (0.14, 1.20)	0.36 (0.17, 0.76)**	0.43 (0.16, 1.14)	0.50 (0.28, 0.88)*	
Yes	1.00	0.50 (0.37, 0.68)***	0.78 (0.44, 1.37)	0.47 (0.32, 0.70)***	0.33 (0.21, 0.51)***	0.68 (0.50, 0.93)*	
DM							0.879
No	1.00	0.39 (0.27, 0.55)***	0.51 (0.26, 0.97)*	0.37 (0.24, 0.57)***	0.31 (0.19, 0.53)***	0.51 (0.37, 0.73)***	
Yes	1.00	0.57 (0.37, 0.88)*	1.02 (0.46, 2.27)	0.594 (0.33, 1.06)	0.42 (0.23, 0.79)**	1.00 (0.64, 1.56)	
BMI							0.019
< 30	1.00	0.33 (0.23, 0.49)***	0.51 (0.26, 1.00)	0.39 (0.23, 0.64)***	0.26 (0.15, 0.44)***	0.50 (0.35, 0.72)***	
≥ 30	1.00	0.55 (0.37, 0.81)**	1.07 (0.51, 2.24)	0.49 (0.30, 0.79)**	0.41 (0.23, 0.74)**	0.85 (0.57, 1.26)	

Analyses was adjusted for age, gender, education level, race/ethnicity, marital status, family poverty-income ratio, hypertension, diabetes mellitus, smoker, alcohol user, body mass index, physical activity, total energy intake, high-density lipoprotein-cholesterol, total cholesterol, triglyceride, uric acid, creatinine, blood urea nitrogen, and hemoglobin when they were not strata variables.

*CVD* cardiovascular disease, *DM* diabetes mellitus, *BMI* body mass index, *OR* odd ratio, *CI* confidence interval.

*P < 0.05, **P < 0.01, ***P < 0.001.

## Discussion

With the aging of the population, the incidence of cardiovascular events is gradually increasing in the elderly^13^. Meanwhile, various risk factors such as unhealthy lifestyle and environmental pollution also make the incidence of cardiovascular events in the young population gradually increased^14^. The most important way to prevent CVD is to promote a healthy lifestyle throughout life, besides, drug control is another important method revealing from the ACC/AHA guideline^15^. Thus, it is of great importance to focus on the primary drug prevention of cardiovascular events in the general population.

As the most commonly used drugs in cardiovascular system, several consensuses have acknowledged the benefits of aspirin in CVD primary prevention, especially its prominent function in reducing the risk of nonfatal myocardial infarction and stroke^16^, meanwhile, the use of aspirin will also lead to the increased incidence of bleeding, as shown by a large-scale meta-analysis^17^. Gaziano et al. constructed a large, randomized, multicenter clinical trial and discovered that based on the low CVD risk participants, the CVD events rate was much lower than expected when the participants were administrated with aspirin, which illustrated the benefits of aspirin in CVD primary prevention^18^. Statins are the first choice for reducing high blood cholesterol, a risk factor for CVD events, mainly due to the reduction of cholesterol biosynthesis^19^. Taylor et al. summarized that people without evidence of CVD treated with statins showed reductions in all-cause mortality, major vascular events and revascularizations with no excess of adverse events^20^. Researchers have constructed several clinical trials to evaluate whether the combination use of aspirin and statins could be beneficial for patients. Athyros et al. found that comparing with statins or aspirin used alone, combination usage of aspirin and statin significantly reduced the CVD events in dyslipidaemic patients^21^. A Korean national cohort study^22^ evaluating the efficacy of aspirin and statins in primary prevention of cardiovascular mortality showed that the combination use of aspirin and statins could benefit the participants.

Statin therapy can improve peripheral atherosclerosis and reverse atherosclerotic plaques. However, rare research focused on the choice of statins with the combination use of aspirin in CVD primary prevention. Here, based on the large-scale population integrated from NHANES database, we reported that the combination of aspirin and rosuvastatin could remarkably reduce the incidence of total and individual CVDs comparing with the combination use of aspirin and other kinds of statins, revealing the advantage of rosuvastatin. Rosuvastatin is the latest and most potent statin currently on the market. Compared with other statins, rosuvastatin is a fully synthetic statin which acts by interfering with the endogenous synthesis of cholesterol through competitively inhibiting the 3-hydroxy-3-methylglutaryl coenzyme^23^. In primary prevention populations, all statins reduced the risk of all-cause, and CVD mortality, but some harm risks also increased. Different statin types had different benefit-harm profiles. A drug-level network meta-analysis showed that atorvastatin and rosuvastatin reduced CVD events most effectively^24^. And, Liping also found that the risk of adverse reactions was not increased with rosuvastatin compared with atorvastatin^25^. In addition, in an intermediate-risk, ethnically diverse population without CVD, rosuvastatin at 10 mg per day significantly decreased cardiovascular events compared with placebo^26^. Compared to other statins, rosuvastatin has been shown to reduce LDL cholesterol more effectively than most other statins^27^ and has the best efficacy in reducing total cholesterol and low-density lipoprotein cholesterol (LDL-C), and also increases HDL-C more than atorvastatin^28^. In East Asian patients with hypercholesterolemia, Zhang and his team revealed that rosuvastatin was more effective than atorvastatin^29^. However, Perez-Calahorra has found that there is no difference in ASCVD recurrence between high doses of rosuvastatin and atorvastatin^30^.

### Strengths and limitations

Strengths of this study included the relatively large sample size to investigate the use of aspirin in conjunction with various statins for CVD prevention. Despite the fact that our research found that combining aspirin and statins is advantageous to the cardiovascular system in the general population. The most important option for primary prevention is still lifestyle modification. More specific group classifications, which NHANES did not offer, should be explored, such as whether individuals were at low, moderate, or high CVD risk, to better provide drug usage evidence. To evaluate the advantages, the adverse effects of aspirin and different stains should also be considered.

## Conclusion

Our results demonstrate that the combination use of aspirin and statins are more effective than using aspirin alone in CVD prevention based on the large-scale U.S. general population. Moreover, rosuvastatin showed more remarkable effects in total and individual CVDs prevention comparing with other statins when combining use with aspirin.

## Data Availability

Survey data is available for data consumers and researchers all across the globe on the internet (https://www.cdc.gov/nchs/nhanes/).

## References

[1] Al-Mallah MH, Sakr S, Al-Qunaibet A (2018). Cardiorespiratory fitness and cardiovascular disease prevention: An update. Curr. Atheroscler. Rep..

[2] Rhee TG, Kumar M, Ross JS, Coll PP (2021). Age-related trajectories of cardiovascular risk and use of aspirin and statin among US adults aged 50 or older, 2011–2018. J. Am. Geriatr. Soc..

[3] Hybiak J, Broniarek I, Kiryczyński G, Los LD, Rosik J, Machaj F (2020). Aspirin and its pleiotropic application. Eur. J. Pharmacol..

[4] Singal AK, Karthikeyan G (2019). Aspirin for primary prevention: Is this the end of the road?. Indian Heart J..

[5] Dietrich E, Davis K (2014). A statin a day to keep the doctor away? Comparing aspirin and statins for primary prevention of cardiovascular disease. Ann. Pharmacother..

[6] Ricciotti E, FitzGerald GA (2021). Aspirin in the prevention of cardiovascular disease and cancer. Annu. Rev. Med..

[7] Faubion SS, Kapoor E, Moyer AM, Hodis HN, Miller VM (2019). Statin therapy: Does sex matter?. Menopause.

[8] Kadoglou NPE, Stasinopoulou M (2021). How to use statins in secondary prevention of atherosclerotic diseases: From the beneficial early initiation to the potentially unfavorable discontinuation. Cardiovasc. Drugs Ther..

[9] Curtin, L. R. *et al*. *The National Health and Nutrition Examination Survey: Sample Design, 1999–2006. Vital and Health Statistics Series 2, Data Evaluation and Methods Research*, Vol. 155, 1–39 (2012). 22788053

[10] Zipf, G. *et al*. *National Health and Nutrition Examination Survey: Plan and Operations, 1999–2010. Vital and Health Statistics Series 1, Programs and Collection Procedures*, Vol. 56, 1–37 (2013). 25078429

[11] Bao W, Liu B, Simonsen DW, Lehmler HJ (2020). Association between exposure to pyrethroid insecticides and risk of all-cause and cause-specific mortality in the general US adult population. JAMA Intern. Med..

[12] Liao S, Zhang J, Shi S, Gong D, Lu X, Cheang I (2020). Association of aldehyde exposure with cardiovascular disease. Ecotoxicol. Environ. Saf..

[13] Noale M, Limongi F, Maggi S (2020). Epidemiology of cardiovascular diseases in the elderly. Adv. Exp. Med. Biol..

[14] Andersson C, Vasan RS (2018). Epidemiology of cardiovascular disease in young individuals. Nat. Rev. Cardiol..

[15] Arnett DK, Blumenthal RS, Albert MA, Buroker AB, Goldberger ZD, Hahn EJ (2019). 2019 ACC/AHA guideline on the primary prevention of cardiovascular disease: A report of the American College of Cardiology/American Heart Association Task Force on Clinical Practice Guidelines. Circulation.

[16] Richman IB, Owens DK (2017). Aspirin for primary prevention. Med. Clin. N. Am..

[17] Zheng SL, Roddick AJ (2019). Association of aspirin use for primary prevention with cardiovascular events and bleeding events: A systematic review and meta-analysis. JAMA.

[18] Gaziano JM, Brotons C, Coppolecchia R, Cricelli C, Darius H, Gorelick PB (2018). Use of aspirin to reduce risk of initial vascular events in patients at moderate risk of cardiovascular disease (ARRIVE): A randomised, double-blind, placebo-controlled trial. Lancet.

[19] Kazi DS, Penko JM, Bibbins-Domingo K (2017). Statins for primary prevention of cardiovascular disease: Review of evidence and recommendations for clinical practice. Med. Clin. N. Am..

[20] Taylor F, Huffman MD, Macedo AF, Moore TH, Burke M, Davey Smith G (2013). Statins for the primary prevention of cardiovascular disease. Cochrane Database Syst. Rev..

[21] Athyros VG, Mikhailidis DP, Papageorgiou AA, Bouloukos VI, Pehlivanidis AN, Symeonidis AN (2005). Effect of statins and aspirin alone and in combination on clinical outcome in dyslipidaemic patients with coronary heart disease. A subgroup analysis of the GREACE study. Platelets.

[22] Lee CJ, Oh J, Lee SH, Kang SM, Choi D, Kim HC (2017). Efficacy of aspirin and statins in primary prevention of cardiovascular mortality in uncomplicated hypertensive participants: A Korean national cohort study. J. Hypertens..

[23] Cortese F, Gesualdo M, Cortese A, Carbonara S, Devito F, Zito A (2016). Rosuvastatin: Beyond the cholesterol-lowering effect. Pharmacol. Res..

[24] Yebyo HG, Aschmann HE, Kaufmann M, Puhan MA (2019). Comparative effectiveness and safety of statins as a class and of specific statins for primary prevention of cardiovascular disease: A systematic review, meta-analysis, and network meta-analysis of randomized trials with 94,283 participants. Am. Heart J..

[25] Liping Z, Xiufang L, Tao Y, Baomin Z, Houshuai T (2018). Efficacy comparison of rosuvastatin and atorvastatin in the treatment of atherosclerosis and drug safety analysis. Pak. J. Pharm. Sci..

[26] Yusuf S, Bosch J, Dagenais G, Zhu J, Xavier D, Liu L (2016). Cholesterol lowering in intermediate-risk persons without cardiovascular disease. N. Engl. J. Med..

[27] Wander GS, Hukkeri MYK, Yalagudri S, Mahajan B, Panda AT (2018). Rosuvastatin: Role in secondary prevention of cardiovascular disease. J. Assoc. Phys. India.

[28] Efthimiadis A (2008). Rosuvastatin and cardiovascular disease: Did the strongest statin hold the initial promises?. Angiology.

[29] Zhang L, Zhang S, Yu Y, Jiang H, Ge J (2020). Efficacy and safety of rosuvastatin vs atorvastatin in lowering LDL cholesterol: A meta-analysis of trials with East Asian populations. Herz.

[30] Perez-Calahorra S, Laclaustra M, Marco-Benedi V, Pinto X, Sanchez-Hernandez RM, Plana N (2019). Comparative efficacy between atorvastatin and rosuvastatin in the prevention of cardiovascular disease recurrence. Lipids Health Dis..

